# Cerebral malaria induced by *plasmodium falciparum*: clinical features, pathogenesis, diagnosis, and treatment

**DOI:** 10.3389/fcimb.2022.939532

**Published:** 2022-07-25

**Authors:** Xiaonan Song, Wei Wei, Weijia Cheng, Huiyin Zhu, Wei Wang, Haifeng Dong, Jian Li

**Affiliations:** ^1^ School of Basic Medical Sciences, Hubei University of Medicine, Shiyan, China; ^2^ Beijing School of Chemistry and Bioengineering, University of Science and Technology Beijing, Beijing, China; ^3^ Key Laboratory of National Health Commission on Technology for Parasitic Diseases Prevention and Control, Jiangsu Provincial Key Laboratory on Parasites and Vector Control Technology, Jiangsu Institute of Parasitic Diseases, Wuxi, China; ^4^ Guangdong Key Laboratory for Genome Stability and Human Disease Prevention, Department of Biochemistry and Molecular Biology, School of Medicine, Shenzhen University, Shenzhen, China

**Keywords:** cerebral malaria, *Plasmodium falciparum*, neurological damage, clinical manifestation, blood–brain barrier, clinical treatment

## Abstract

Cerebral malaria (CM) caused by *Plasmodium falciparum* is a fatal neurological complication of malaria, resulting in coma and death, and even survivors may suffer long-term neurological sequelae. In sub-Saharan Africa, CM occurs mainly in children under five years of age. Although intravenous artesunate is considered the preferred treatment for CM, the clinical efficacy is still far from satisfactory. The neurological damage induced by CM is irreversible and lethal, and it is therefore of great significance to unravel the exact etiology of CM, which may be beneficial for the effective management of this severe disease. Here, we review the clinical characteristics, pathogenesis, diagnosis, and clinical therapy of CM, with the aim of providing insights into the development of novel tools for improved CM treatments.

## Introduction

Malaria, a mosquito-transmitted infectious disease caused by *Plasmodium* species, remains a significant public health concern globally (XP D, L S, 2021). In 2020, 241 million malaria cases were estimated, and 627,000 deaths occurred, with 77% found among children under five years of age (Jiang et al., 2021). Currently, five *Plasmodium* spp. are reported to infect humans, including *P. falciparum*, *P. ovale*, *P. vivax*, *P. malariae*, and *P. knowlesi*. As we know, *P. falciparum* is considered the most severe species and the primary cause of mortality, notably in young children (Su and Wu, 2021). Cerebral malaria (CM) is a fatal neurological complication of *P. falciparum* malaria (Luzolo and Ngoyi, 2019), and children aged under 3 years and pregnant women are most susceptible (World Health Organization, 2021). The mortality of CM is estimated to be 20% in children and 30% in adults (Solomon et al., 2014). Furthermore, 15-20% of survivors suffer long-term neurological sequelae, such as hemiplegia, ataxia, speech disorders, and epilepsy, resulting in lifelong neurological deficits and even death (Birbeck et al., 2010). Hereby, we review the clinical manifestations, pathogenesis, diagnosis, and treatment of CM so as to provide insights into the management of CM.

## Clinical manifestations of CM

CM is clinically characterized as a diffuse encephalopathy with a history of fever for 2 to 3 days, subsequent seizures, and loss of consciousness (coma). Previous studies have demonstrated substantial differences in the clinical manifestations of CM between children and adults (**Table 1**). Although this may be attributed to immune status and age, there are still many questions that remain to be answered (Olliaro, 2008; Sahu et al., 2021). Pediatric CM usually manifests with coma, seizures, and severe anemia, while renal failure and respiratory distress rarely occur in African children (Waller et al., 1995; Newton et al., 2000). Nevertheless, adult CM is frequently associated with multiple organ complications, including central nervous system (CNS) and liver dysfunction, respiratory failure, and acute kidney failure (Mishra et al., 2007; Wassmer et al., 2015).

**Table 1:** Clinical manifestations of pediatric and adult cerebral malaria.

Clinical features		Children	Adults
Preceding symptoms		Fever, failure to eat or drink, vomiting and cough, and convulsions (Molyneux et al., 1989).	General malaise, head, back, and limb pain, dizziness, anorexia, nausea, vomiting, and mild diarrhea (NIH (2014)).
Neurological system	Coma	It develops rapidly, often after a seizure, and lasts for 1 to 2 days, reversible (Genton et al., 1997; Newton et al., 2000).	Develops gradually following delirium, Disorientation, and agitation over 2 to 3 days or follows a generalized seizure, lasts longer (2 days) (Idro et al., 2005).
	Nerve reflex	More common (Waller et al., 1995).	Rare.
	Neurological impairments	Ataxia (43%), hemiplegia (39%), speech disorders (39%) and blindness (30%). Other sequelae include behavioral disturbances, hypotonia, generalized spasticity, and a variety of tremors (van Hensbroek et al., 1997).	Psychosis, psychosis, ataxia, transitory cranial nerve palsies, mononeuritis multiplex, polyneuropathy, extrapyramidal and extrapyramidal tremors, and other cerebellar signs (van Hensbroek et al., 1997).
Motor system	Seizures	High incidence, frequently mostly partial motor (Crawley et al., 1996).	Low incidence, generalized seizures frequently, less focal (Idro et al., 2005).
	Status epilepticus	Usual (Crawley et al., 1996).	Rare (Vespa et al., 1999).
	Abnormal behavior	Hyperactivity, impulsiveness, and inattentiveness or conduct disorders (Birbeck et al., 2010; Idro et al., 2010).	Ataxia of gait, intention tremor, dysmetria, dysdiadochokinesis, nystagmus, and cerebellar dysarthria (Senanayake, 1987).
Systemic complications		Hyponatremia, anemia, hypoglycemia, jaundice, metabolic acidosis, respiratory distress, hepatosplenomegaly, and intracranial pressure (English et al., 1996; English et al., 1997; English et al., 1998; Idro et al., 2005).	Anemia, hypoglycemia, hemoglobinuria, jaundice, shock, renal failure, severe lactic acidosis, abnormal bleeding, pulmonary edema, and adult respiratory distress syndrome, Kussmaul’s breathing (Garg et al., 1999; Hora et al., 2016).
Retinopathy		Retinal whitening, orange or white discoloration of the retinal vessels, retinal hemorrhages, and infrequent papilledema (MacCormick et al., 2014).	Less prominent. Characterized by retinal hemorrhages and retinal whitening, no change in retinal vessel discoloration (NIH (2014)).

## Neurological system

### Seizure

Compared with adults, children have a higher incidence rate of seizures (Postels and Birbeck, 2013). In children, focal motor and generalized tonic–clonic convulsions are the most common clinically detected seizures; however, subtle or subclinical seizures detected with electroencephalography (EEG) are also common (Newton et al., 2000; Postels and Birbeck, 2013). Subtle seizures manifest as nystagmoid eye movements, irregular breathing, excessive salivation, and conjugate eye deviation (Crawley et al., 1996). Most seizures in adult CM patients are generalized seizures; however, focal motor seizures may also occur. Occasionally, the sign of seizure activity is subtle, such as repetitive eye or hand movements, and may be easily overlooked. Subtle seizure activity seems to be more common in children than in adults (Newton et al., 2000). The level of consciousness after a seizure is usually lower than that preceding it. Status epilepticus is unusual in adults, although more than one seizure is frequent (Vespa et al., 1999). Previous studies reported an association between status epilepticus and neurological sequelae among CM patients, which occur in 5-15% of survivors (Brewster et al., 1990), and it has been shown that prolonged seizure activity may damage the brain, causing deficits in both motor and cognitive functions (Stafstrom et al., 1993).

### Coma

Coma usually develops rapidly after seizures among children living in malaria-endemic areas, and consciousness recovers to normal rapidly (within 24-48 h) (Genton et al., 1997). Different disease processes may affect awareness in children with malaria, including convulsions, hypoglycemia, hyperpyrexia, acidosis, severe anemia, and sedative drugs. Although the cause of impaired consciousness or coma remains unclear, it is likely to result from several interacting mechanisms (Newton et al., 2000). Adhesion of malaria parasite-infected red blood cells (iRBCs) reduces microvascular blood flow (Kaul et al., 1998), which may be the cause of organ tissue dysfunction, such as coma. High concentrations of tumor necrosis factor-α (TNF-α) are associated with coma (Kwiatkowski et al., 1990; Kaul et al., 1998). Compared to children, coma gradually develops in adults following drowsiness, disorientation, delirium, and agitation within 2 to 3 days (Kochar et al., 2002). Convulsion leads to the development of a coma and occurs in approximately 15% of adults and 80% of children (Plewes et al., 2018).

## Neurologic features

Abnormal corneal and oculocephalic reflexes (doll’s eye) are likely to occur in children with deep coma. Abnormal plantar reflexes are also detected, and abdominal reflexes are almost invariably absent. In adults with profound coma, corneal and eyelash reflexes are usually intact unless in a state of deep coma, and the pupils are normal. Forcible jaw closure and teeth grinding (bruxism) are commonly seen in CM. Pout reflex usually indicates a “frontal release”; however, the grasp reflex is frequently absent. In addition, increased muscle tone and tendon reflexes are found. CM may elicit ankle and patellar clonus, and extensor plantar responses. Nevertheless, abdominal and cremasteric reflexes are invariably absent ().

### Neurological impairments

CM affects the CNS, and although most survivors have a full recovery, 3-31% of patients still develop neurological deficits and cognitive sequelae (Oluwayemi et al., 2013). The prevalence of neurological deficits is higher in children than in adults, ranging from 6% to 29% at the time of discharge (Idro et al., 2004; Hawkes et al., 2013). Children with CM frequently present long-term neurologic deficits, and episodes of CM imply the development of long-term sequelae in children. In children, the most common sequelae include ataxia, paralysis, paresis, cortical blindness, epilepsy, deafness, behavioral disorders, language disorders, and cognitive impairment (Brewster et al., 1990). Sequelae are less common in adults. During the acute phase of CM, neurologic abnormalities include psychosis, ataxia, transitory cranial nerve palsies or tremor (Peixoto and Kalei, 2013).

## Retinopathy

The characteristic features of retinopathy due to CM include retinal whitening (macula whitening sparing central fovea and peripheral whitening of the fundus), retinal vessel discoloration to pink–orange or white, retinal hemorrhages, and papilledema (Hora et al., 2016). The first two abnormalities are considered specific symptoms of CM. Commonalities between pediatric and adult patients include retinal hemorrhage, a common manifestation but a less distinctive feature. Retinal hemorrhage correlates with disease severity and cerebral hemorrhage in the microvascular dissection of the brain (White et al., 2001). Papilledema is rare in children and adults. Although it is a nonspecific symptom of CM, it reflects increased intracranial pressure and portends a poor prognosis in children (Beare et al., 2004). A prominent difference between children and adults is vessel discoloration. Orange or white discoloration of the retinal vessels has been attributed to the hemoglobinization of stationary erythrocytes infected with mature parasites (Beare et al., 2011). The degree of retinal microvascular damage is comparable to cerebral damage (Beare et al., 2004; Lewallen et al., 2008).

## Non-CNS abnormalities in CM

Systemic complications include anemia (20% to 50% incidence), hypoglycemia (30% incidence), hyponatremia (>50% incidence), jaundice (8% incidence), metabolic acidosis characterized by respiratory distress, and hepatosplenomegaly in children living with CM (White et al., 1987; English et al., 1996; Idro et al., 2005; Maitland and Newton, 2005). Renal failure and pulmonary edema are unusual in children (Newton et al., 1991). CM predominantly manifests as CNS dysfunction in children; however, it is mainly present in multisystem and organ (circulatory, hepatic, coagulation, renal, and pulmonary) dysfunctions in adults (Day et al., 2000; Krishnan and Karnad, 2003).

In adults, anemia is an inevitable consequence of CM and develops exceptionally rapidly. CM has been reported in patients together with pulmonary edema, adult respiratory distress syndrome and hemoglobinuria, and Kussmaul’s breathing occurs with acute renal failure and severe lactic acidosis (Newton and Warrell, 1998). Hypoglycemia occurs in 8% of patients aged 26 to 28 years (White et al., 1983). Other complications included jaundice, shock, abnormal bleeding, and coagulopathy.

## Pathogenesis of CM

**Figure 1:**
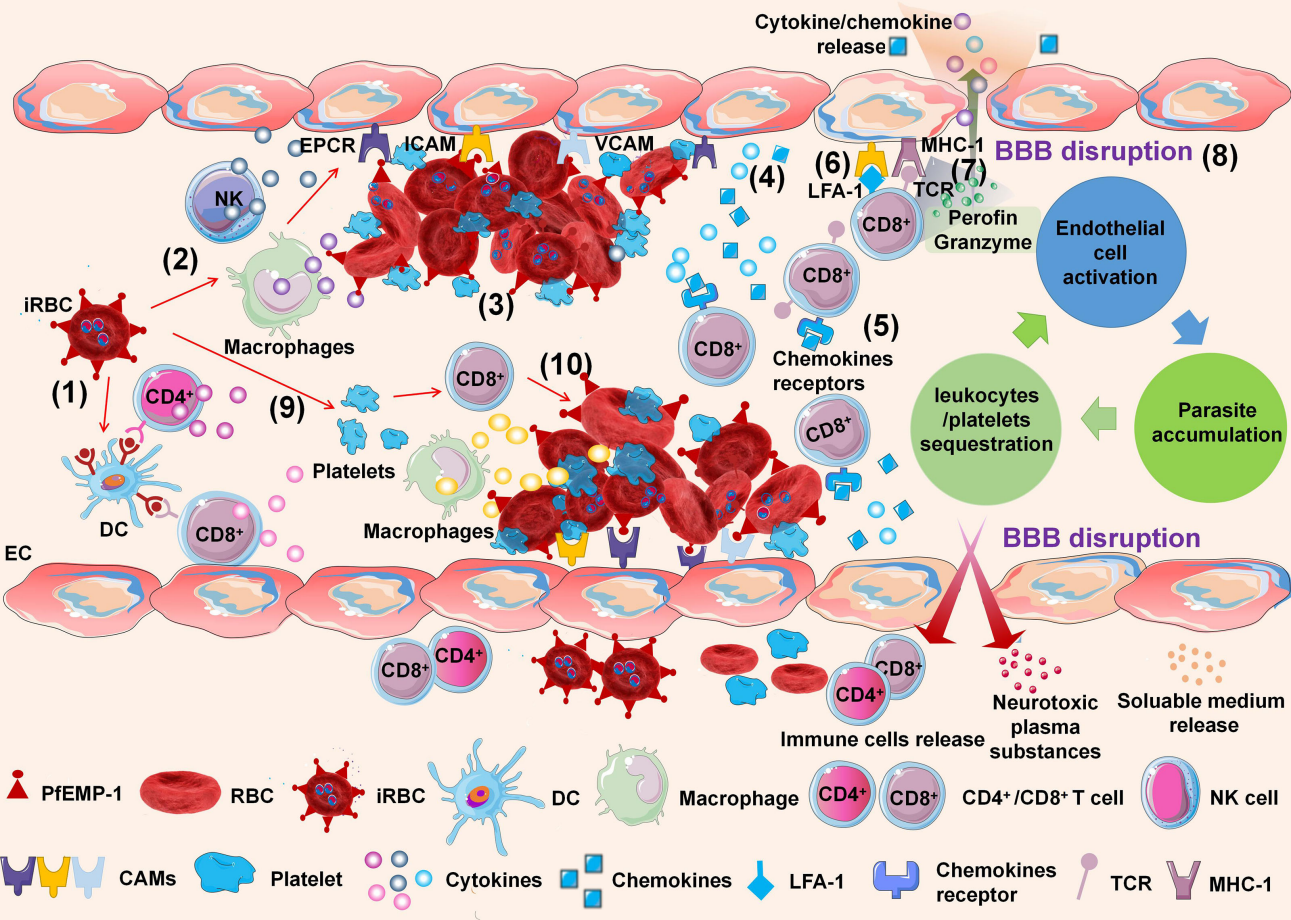
Schematic of experimental cerebral malaria (ECM) pathogenesis. The ECM is initiated by dendritic cells (DCs) processing and presenting infected red blood cell (iRBC) antigens to CD4^+^ and CD8^+^ T cells in the spleen (1). NK cells and macrophages are activated by iRBCs to secrete inflammatory cytokines (2). The iRBCs adhere to endothelial cells (ECs) of the brain microvasculature through the interaction between *P. falciparum* erythrocyte membrane protein-1 (PfEMP-1) of iRBCs and cell adhesion molecules of ECs (3). The adhesion of iRBCs to the cerebral microvascular endothelium is also further accompanied by agglutination to other iRBCs, platelets, white blood cells (WBCs), and the rosetting effect formed by the adhesion of iRBCs and RBCs. ECs are activated by interactions with iRBCs and responses to inflammatory cytokines. Activated ECs promote the upregulation of cell adhesion molecules (CAMs) on brain ECs and release cytokines and chemokines simultaneously (4). Activated CD8^+^ T cells express CXCR3 and CCR5 chemokine receptors, which bind to chemokines such as CXCL9, CXCL10, and CXCL4, inducing T-cell migration to the brain (5). Meanwhile, LFA-1 on CD8^+^ T cells promotes their adhesion to endothelial ICAM-1 (6). Parasitic antigens can be transferred from the vascular lumen to brain ECs. Brain ECs can cross-present parasitic antigens on MHC-1 molecular antigens and bind with antigen receptors (TCRs) on CD8^+^ T cells (7). The interaction induces apoptosis of ECs, leading to the destruction of the BBB (8). Meanwhile, the iRBCs directly activate platelets and stimulate the release of CXCL4. CXCL4 induces the production of TNF by T cells and macrophages, which causes more platelets to adhere to ECs (9). As leukocytes and platelets are recruited and activated, a local proinflammatory cycle ensues, with a positive feedback loop of EC activation, leukocyte/platelet sequestration, and parasite accumulation (10).

Although the pathophysiology of CM has been extensively investigated, the exact pathogenesis remains unclear. Currently, CM is widely accepted as a multifactorial process related to the adhesion and sequestration of iRBCs, immunological responses, endothelial cell (EC) activation, and loss of BBB integrity (Idro et al., 2005). Nevertheless, any of these mechanisms alone fail to explain the pathogenesis of human CM, and they jointly participate in this potentially fatal infection. A mouse model of experimental cerebral malaria (ECM) has been used to simulate and explain the pathogenesis of human CM (**Figure 1**).

## Adhesion and sequestration

Cerebral iRBCs adherence is an indicative marker of CM in adults and children, and it is considered a starting point during the development of CM. Sequestration is thought to be a specific interaction between iRBCs and vascular ECs, which is not limited to brain tissues but also occurs on ECs in different organs, including the lung, kidney, liver, and intestine.

The adhesion of iRBCs to the vascular endothelium is mediated by *P. falciparum* erythrocyte membrane protein 1 (PfEMP1) (Jensen et al., 2020), a specific cell-surface ligand expressed by iRBCs. PfEMP1 belongs to the antigen-variant protein family, and the *var* genes encoding the protein are a large multigene family (Kim, 2012). To date, 60 different *var* genes have been characterized, and *var* gene-encoded proteins have shown dual functions in regulating antigen variation and cell adhesion (Tembo et al., 2014). PfEMP1 contains a host molecule binding domain and binds to several cell adhesion molecules (CAMs) on ECs, such as CD36 (Berendt et al., 1989; Ockenhouse et al., 1989), intercellular adhesion molecule 1 (ICAM-1) (Berendt et al., 1989), vascular adhesion molecule 1 (VCAM-1) (Ockenhouse et al., 1989; Ockenhouse et al., 1992), endothelial protein C receptor (EPCR) (Mohan Rao et al., 2014), thrombospondin, E-selectin (Turner et al., 1994) and chondroitin sulphate A (Rogerson et al., 1995; Fried and Duffy, 1996). Adhesion of iRBCs to the cerebral microvascular endothelium is further accompanied by agglutination to other iRBCs, platelets, white blood cells (WBCs), and rosetting produced by adhesion of iRBCs and uninfected erythrocytes (Fried and Duffy, 1996). Sequestration of iRBCs in microvessels may protect iRBCs from clearance by the spleen. In addition, it weakens the capability of iRBCs and RBCs to denature, leading to blood vessel blockage. Previous studies reported a significant correlation between sequestration of iRBCs in cerebral vessels and coma in CM patients (Silamut et al., 1999; Ponsford et al., 2012; Storm et al., 2019). Taken together, sequestration of iRBCs leads to increased vasoconstriction and vascular obstruction, as well as decreased cerebral blood flow and hypoxia.

## Inflammatory responses

Excessive immune responses and the release of a large number of inflammatory factors play important roles in the pathogenesis of CM (Shikani et al., 2012). The humoral response to malaria parasites includes immune activation of macrophages and lymphocytes (CD8^+^, CD4^+^, natural killer (NK) cells) and activation of monocytes, resulting in accumulation of immune cells in the microvasculature and a systemic inflammatory response secreted by proinflammatory cytokines, including tumor necrosis factor (TNF)-α, interferon (IFN)-γ, and interleukin-1β (IL-1β), which are elevated in an episode of acute CM.

At the early stage of malaria infection, CD4^+^ and CD8^+^ T cells are activated by antigen-presenting cells (APCs) to initiate antimalarial protective cellular immune responses. The chemotaxis of T cells to peripheral cerebral vessels is one of the prominent features of CM. Recruitment of CD8^+^ T cells is the most predominant characteristic (Riggle et al., 2020), and priming of CD4^+^ and CD8^+^ T cells initiates CM in the spleen by dendritic cells (DCs) presenting iRBCs antigens. NK cells and macrophages are activated by iRBCs to release inflammatory cytokines, such as TNF-α, IFN-γ, IL-1β, IL-12 and chemokines (Dunst et al., 2017). Adhesion of iRBCs and the release of inflammatory cytokines can activate brain ECs, triggering ECs to produce chemokines and inflammatory cytokines and upregulate CAM expression. Activation of CD8^+^ T cells results in the expression of chemokine receptors, including CXCR3 and CCR5. Subsequently, chemokine receptors bind to chemokine ligands expressed by ECs to induce CD8^+^ T-cell migration and infiltration into brain ECs. CD11a (LFA-1) on CD8^+^ T cells promotes adhesion to endothelial ICAM-1 (Howland et al., 2015; Dunst et al., 2017), and upregulated expression of CAMs induces increased recruitment of iRBCs, WBCs, and platelets in brain capillaries, which enhances cerebral microvascular sequestration (McEver, 2001; Shikani et al., 2012). The rupture of iRBCs releases merozoites, which are endocytosed by ECs and then cross-presented on major histocompatibility complex class 1 (MHC-1) molecules. MHC-1 binds to antigen receptors (TCRs) on effector CD8^+^ T cells to activate CD8^+^ T cells (Howland et al., 2013). Activated CD8^+^ T cells release perforin, granzyme-B, and chemokines, triggering NK cells and macrophages to migrate toward the brain. Immune cell accumulation and perforin release induce apoptotic signaling in ECs and alter the tight junctions of ECs, resulting in EC dysfunction and increased cerebral vascular permeability (Yañez et al., 1996; Belnoue et al., 2002; Haque et al., 2011). Disruption of BBB integrity frequently results in perivascular space enlargement, edema formation, and increased intracranial pressure, eventually resulting in death.

## Activation of vascular ECs

Activation of microvascular ECs is a central component of brain microvascular pathology, resulting from the sequestration of iRBCs on the surface of vascular ECs and systematic release of inflammatory cytokines (Siddiqui et al., 2020). Activated ECs are well characterized by aggravation of brain microvascular sequestration, breakdown of tight junctions, and initiation of coagulation cascading reactions.

EPCR, a host receptor involved in anticoagulation and endothelial protection, has been identified as a receptor of PfEMP1 (Turner et al., 2013). It is speculated that EPCR mediates iRBCs sequestration and participates in thrombin-induced disruption of the BBB. EPCR plays a crucial role in stabilizing ECs by activating activated protein C, an inhibitor of thrombin production that prevents EC activation (Mohan Rao et al., 2014). In CM, some variants of the *Plasmodium* adhesins PfEMP-1 (called DC8 and DC13) preferentially bind to EPCR. Upon binding to EPCR, iRBCs reduce the level of available EPCR binding sites and block the activation of activated protein C by EPCR (Shabani et al., 2017). Induction of the coagulation pathway by reducing the synthesis of EPCR and activated protein C leads to increased thrombin production and EC activation, as well as decreased protective effects of ECs.

Platelets are considered effector cells of the hemostasis system and contribute to CM. It is actively involved in sequestration, inflammation, and coagulation dysfunction and is identified as their joint point (Cox and McConkey, 2010). Platelets bind to iRBCs (agglutination) and ECs *via* adhesion receptors (CD36, ICAM-1, P-selectin). In addition, platelets promote immune activation by binding Toll-like receptors to parasite-derived molecules, expressing chemokine receptors, and releasing cytokines, chemokines, and other immunomodulatory molecules. All these activated cells (ECs, platelets, monocytes) release microparticles (TNF-α, IFN-γ) (Combes et al., 2004). Taken together, microparticles alter EC functions and are regarded as proinflammatory factors and cellular activation markers.

## BBB disruption

The BBB is a semipermeable membrane that separates the peripheral blood from the cerebral parenchyma and maintains balance by protecting the brain from potentially harmful blood pathogens and chemicals. The BBB consists of the microvascular endothelium, pericytes, microglia, astrocyte end-feet, neurons, and basement membrane. Microvascular ECs have tight junctions that impede the passive paracellular diffusion of small and large molecules (Abbott et al., 2010; Moura et al., 2017).

**Figure 2:**
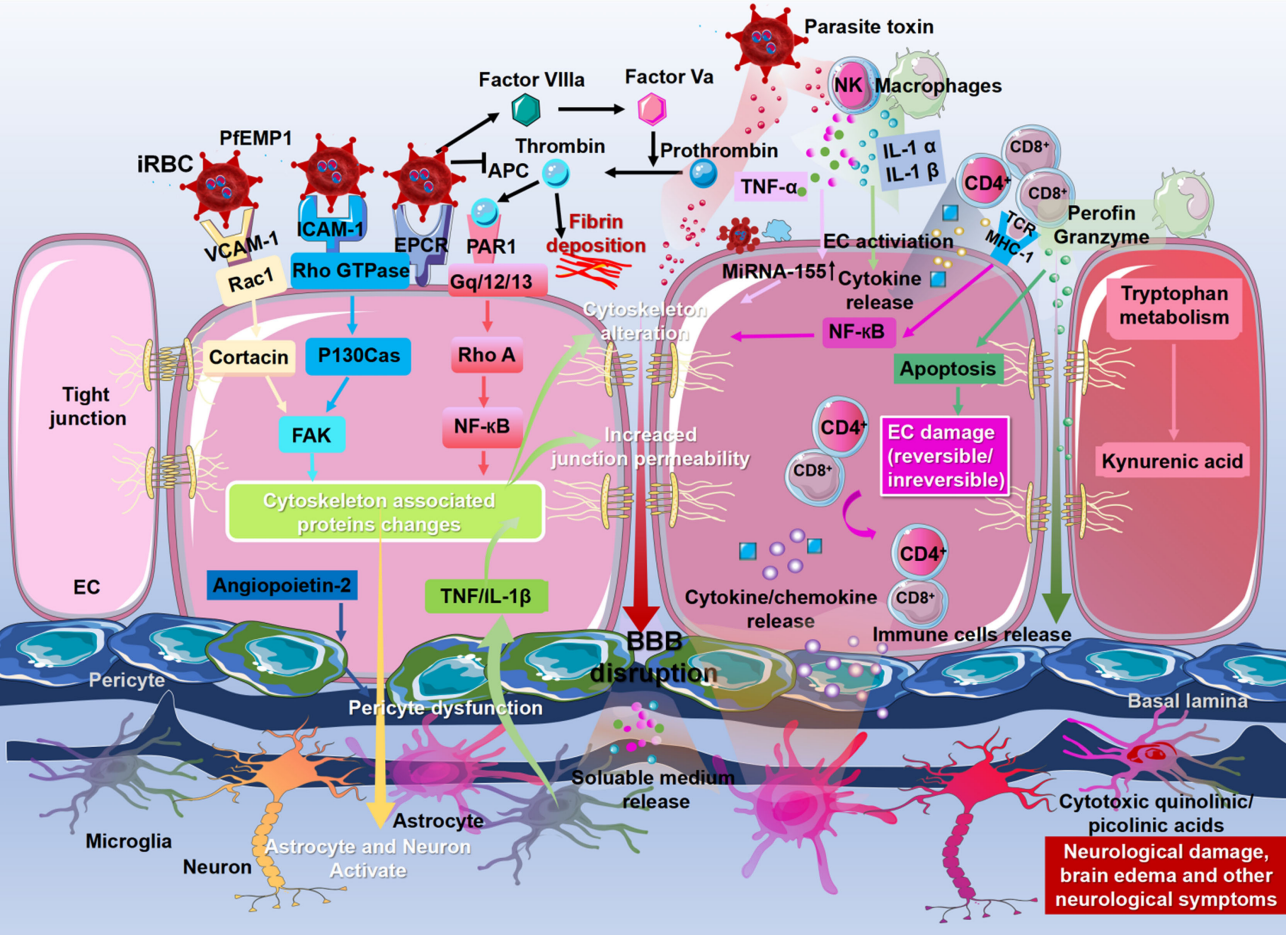
Molecular mechanisms of blood–brain barrier dysfunction. The binding of *P. falciparum* erythrocyte membrane protein-1 (PfEMP-1) to the receptors on the ECs, including ICAM-1, VCAM-1, and EPCR, may trigger multiple signaling pathways in ECs, leading to the change to cytoskeleton-associated proteins, ultimately resulting in the disruption of the BBB. Meanwhile, signaling pathways triggered by PfEMP1 lead to activation and injury of astrocytes, microglia, neurons, and perivascular macrophages and initiate the process of neuropathological injury. The binding of PfEMP1 to EPCR fosters the activation of tissue factors Va and VIIIa, thereby disrupting the anticoagulant pathway. Activation of these tissue factors results in thrombin generation, leading to fibrin deposition. Microglia also disrupt the BBB by producing TNF and IL-1β. Astrocytes retract their end feet from ECs, resulting in reduced vascular wrapping. Angiopoietin-2 produced by ECs also leads to reduced vascular wrapping by inducing pericyte dysfunction. The iRBCs stimulate leukocytes to release inflammatory cytokines (TNF-α, IL-1α, IL-1β) by releasing parasitic toxins. These cytokines disrupt BBB integrity by altering tight junctions and activating ECs to release chemokines (CCL2, CCL4, CXCL4, CXCL8, and CXCL10), which promote leukocyte accumulation, including CD4^+^ T cells and CD8^+^ T cells. Infiltrated leukocytes induce EC apoptosis through granzyme B and perforin-mediated cytotoxicity. Granzyme B and perforin directly induce neuronal cell death. Adhesion of iRBCs, leukocytes, and platelets to ECs also causes EC damage and irreversible changes. Due to the increased permeability of the BBB, cytokines, chemokines, immune cells, and plasma factors flood into the brain parenchyma and activate neurons and astrocytes, resulting in nerve injury and neurological sequelae. Kynurenic acid produced by macrophages and ECs during tryptophan metabolism is further converted into cytotoxic quinoline, which plays a vital role in stromal cells and microglia. These molecules induce the disruption of the BBB.

Binding of PfEMP1 to receptors on ECs, including ICAM-1, VCAM-1, and EPCR, may trigger multiple signaling pathways in ECs, leading to reorganization of the tight junction complex and ultimately resulting in BBB leakage. ICAM-1 induces endothelial cytoskeletal remodeling *via* Rho-dependent phosphorylation of cytoskeleton-associated proteins, including FAK, paxillin, p130Cas, and cortactin, thereby promoting BBB opening (Wittchen, 2009). In addition, VCAM-1 cross-linking results in the activation of Rac1 signaling, which induces the attenuation of tight junctions through Rho-dependent induction of stress fibers. Binding of PfEMP1 to EPCR fosters activation of tissue factors Va and VIIIa, thereby disrupting the anticoagulant pathway. Activation of these tissue factors results in thrombin generation, leading to fibrin deposition. In addition, PfEMP1 binding to EPCR activates the Rho A and NF-κB pathways through thrombin-mediated cleavage of PAR1, which induces a proinflammatory response, leading to BBB disruption (Bernabeu and Smith, 2017; Kessler et al., 2017). Microglia also disrupt the BBB by producing TNF and IL-1β. Adhesion of iRBCs, leukocytes, and platelets to ECs also causes EC damage and irreversible changes (Nishanth and Schlüter, 2019). iRBCs stimulate leukocytes (monocytes, NK cells) to release inflammatory cytokines (TNF-α, IL-1α, IL-1β) by releasing parasitic toxins (Medana and Turner, 2006; Nishanth and Schlüter, 2019). TNF-α upregulates miR-155 expression in ECs, leading to dysfunction of BBB integrity by altering tight junctions (Barker et al., 2017). IL-1α and IL-1β activate ECs to release the chemokines CCL2, CCL4, CXCL8, and CXCL10, which promote leukocyte accumulation (Dunst et al., 2017), and infiltrated leukocytes induce EC apoptosis through granzyme-B and perforin-mediated cytotoxicity (Rénia et al., 2012). CD8^+^ T cells directly induce neuronal cell death through their cytotoxic function and activation of neurons. Due to increased BBB permeability, cytokines, chemokines, immune cells, and plasma factors enter the brain parenchyma and activate neurons and astrocytes, resulting in nerve injury and neurological sequelae (Schiess et al., 2020). Kynurenic acid produced by macrophages and ECs during tryptophan metabolism is further converted into cytotoxic quinoline (Bosco et al., 2003; Medana et al., 2003; Guillemin, 2012). All othese molecules induce disruption of the BBB (**Figure 2**).

Recently, multiomics platforms, including genomics, transcriptomics, proteomics and metabolomics, have been widely used to unravel the underlying pathogenesis of cancer and design therapeutic strategies (Nam et al., 2021). To date, there has been no combined use of multiomics approaches for CM studies, which has inspired the joint analysis of individual omics data. Analysis of DNA markers, RNA transcripts, proteins, and metabolites generated during the progression of CM contributes to understanding CM pathogenesis, which facilitates the precise diagnosis of CM and the discovery of novel therapeutic targets.

## Diagnosis of CM

Diagnosis is central to malaria control, and early diagnosis is one of the crucial factors affecting the prognosis of CM. Unfortunately, there is no gold standard for the diagnosis of CM because of its complex and nonspecific clinical manifestations. Currently, the primary clinical symptoms that are available for CM diagnosis include (1) nonarousal coma (no local responses to pain) that persists for more than six hours after experiencing a generalized convulsion; (2) presence of asexual forms of *P. falciparum* on both thick and thin blood smears; and (3) exclusion of other causes of encephalopathy. To improve the accuracy of CM diagnosis, state-of-the-art cerebral imaging tools are available to assist the diagnosis of CM (**Table 2**).

**Table 2:** Advantages and disadvantages of different approaches for the diagnosis of cerebral malaria.

Diagnostic approaches			Advantages and disadvantages
Imaging approaches	Malaria retinopathy	Fundoscopy	Advantage: relatively low cost and simple, accurate distinction between malarial and nonmalarial comas (Essuman et al., 2010; MacCormick et al., 2014). Disadvantage: requiring trained ophthalmologists and expensive equipment, subject to environmental conditions (Abu Sayeed et al., 2011).
		Optical coherence tomography (OCT)	Advantage: requiring qualitative and quantitative evaluation, noninvasive nature, and high-resolution output (Spaide et al., 2018). Disadvantage: High cost as well as practical issues (Sahu et al., 2015).
		Teleophthalmology	Inexpensive, portable, require little additional training, and suitable for bedside patients in a variety of settings (Salongcay and Silva, 2018).
		Fluorescein fundus angiography (FFA)	Advantage: Reflect the integrity of retinal blood perfusion and blood–retinal barrier by intraretinal fluorescein, and high-resolution digital imaging (Maude et al., 2011). Disadvantage: Large size, bulky and inconvenient to use (Maude et al., 2011).
	Electroencephalography (EEG) and Micro-EEG	EEG	Advantage: Useful, noninvasive, and relatively inexpensive diagnostic tests make it possible to detect delayed cerebral malaria sequelae (Sahu et al., 2015). EEG abnormalities in cerebral malaria patients are manifested by diffuse slowing, atypical sleep elements (fusiform and parietal waves), and epileptiform activity (Postels et al., 2018). Disadvantage: Require continuous postdischarge follow-up assessment.
		Micro-EEG	Miniature, portable, easier continuous recording after patient discharge (Grant et al., 2014),
	Other		Magnetic resonance imaging (MRI) (Mohanty et al., 2017), computed tomography (CT) (Mohanty et al., 2011), intravital microscopy (IVM) (Volz, 2013), and *in vivo* bioluminescent imaging devices (Franke-Fayard et al., 2006).
Biomarkers			High levels of soluble ICAM-1 (Ramos et al., 2013), decreased Ang-1 and increased Ang-2 and Ang-2/Ang-1 (Conroy et al., 2012; Eisenhut, 2012), the elevation of specific smooth muscle proteins in plasma, including carbonic anhydrase III (CA3), creatine kinase (CK), creatine kinase muscle (CKM), and myoglobin (MB) (Bachmann et al., 2014), enhanced plasma levels of CXCL10 and CXCL4 (Wilson et al., 2011). Hsa-miR-3158-3p represents a promising biomarker candidate for CM prognosis (Gupta et al., 2021) and the relative expression levels of miR-19a-3p, miR-19b-3p, miR-146a, miR-193b, miR-467a, miR-27a, and miR-146a may be associated with CM (Martin-Alonso et al., 2018; Wah et al., 2019; Assis et al., 2020).

### Malarial retinopathy

The presence of malarial retinopathy facilitates the improvement in the specificity for the clinical diagnosis of CM and offers strong evidence for CM diagnosis in both adults and children (MacCormick et al., 2014). In pediatric patients, the degree of retinal microcirculation is comparable to that of the brain, making it an easily observable surrogate marker to assess the severity of cerebral pathology during CM (Bearden, 2012). It has been shown that malarial retinopathy presents 100% specificity and 95% sensitivity for the detection of CM, with autopsy as the diagnostic gold standard (Beare et al., 2006).

### Fundoscopy

Fundoscopy is a relatively low-cost and simple technique for the detection of retinopathy, which allows accurate differentiation between malarial and nonmalarial comas. The diagnosis of malarial retinopathy depends on the presence of peripheral retinal whitening, orange and white discoloration of retinal vessels, white-centered hemorrhages, and mild papilledema. The unique retinopathy of patchy retinal whitening and focal changes in vascular color are highly specific for CM diagnosis (Beare et al., 2006; MacCormick et al., 2014). In addition, retinal hemorrhage is a common but less distinctive feature, while papilledema is not specific to CM and is unavailable for CM diagnosis alone.

### Optical coherence tomography

OCT is an *in vivo* imaging tool that detects retinal changes and is feasible for qualitatively and quantitatively evaluating high-resolution cross-sectional retinal images, papilla of the optic nerve, and even retinal nerve fiber layer thickness (Spaide et al., 2018). OCT is a noninvasive, high-resolution measure; however, this technique fails to diagnose malarial retinopathy.

### Teleophthalmology

The introduction of fundoscopy improves the accuracy of CM diagnosis; however, it requires well-trained ophthalmologists and expensive equipment, which restrains its applications in resource-limited settings (Abu Sayeed et al., 2011). To overcome these problems, an innovative approach, teleophthalmology, has emerged for retinal assessment (Salongcay and Silva, 2018). This technique uses a simple and inexpensive portable fundus camera to capture images by well-trained professionals, and then, the images are transferred to ophthalmologists for rapid diagnosis. Teleophthalmology requires little additional training, minimizes healthcare-seeking inconvenience and is feasible in various settings (Maude et al., 2011).

### Fluorescein fundus angiography

With improvements in optical technology and high-resolution digital imaging, FFA has been extensively used by ophthalmologists across the world. FFA measures the integrity of retinal blood perfusion and the blood–retinal barrier by observing a map of the intraretinal fluorescein. CM patients have nonperfusion in the central retina and extensive nonperfusion in the peripheral retina (Glover et al., 2010). Nevertheless, FFA requires a bulky tabletop retinal camera, whose weight and stillness make it difficult to capture clear images from conscious CM patients.

### EEG and micro-EEG

#### EEG

EEG pulses are recorded by measuring voltage fluctuations caused by ionic currents within the neural tissues. This noninvasive technique has made it possible to detect delayed CM sequelae (Sahu et al., 2015), including neurological disorders such as status epilepticus. CM patients’ EEG abnormalities manifest as diffuse slowing, atypical sleep elements (fusiform and parietal waves), and epileptiform activity (Postels et al., 2018).

#### Micro-EEG

Although EEG is a noninvasive and relatively inexpensive diagnostic method, a significant limitation is continuous follow-up assessment of brain activity after discharge from the hospital. To address this concern, micro-EEG, a miniature, wireless, and battery-powered portable headset, was developed, and this device achieved a comparable accuracy for the diagnosis of status epilepticus with standard EEG systems (Grant et al., 2014). This new tool facilitates the recording of brain activity after discharge from the hospital and may provide an option for CM diagnosis.

In addition, other imaging tools, including magnetic resonance imaging (MRI) (Grant et al., 2014; Sahu et al., 2021), computed tomography (CT) (Mohanty et al., 2011; Sahu et al., 2021), intravital microscopy (IVM) (Volz, 2013), and *in vivo* bioluminescent imaging (Franke-Fayard et al., 2006), may serve as additional diagnostic approaches for CM.

## Biomarkers

In addition to imaging tools, biomarkers have been extensively used for the rapid diagnosis of CM. Soluble ICAM-1, which is strongly associated with CM, was reported to be upregulated in the brain microvasculature (Ramos et al., 2013). The soluble EPCR (sEPCR) level at admission is positively correlated with CM and malaria-related mortality, and admission sEPCR was identified as an early biomarker of prognosis among CM patients (Ramos et al., 2013). Angiopoietin-1 (Ang-1) and Ang-2 have been characterized as mediators of endothelial activation and integrity, and Ang-1 maintains vascular quiescence, while Ang-2 displaces Ang-1 upon endothelial activation and sensitizes cells to become responsive to subthreshold concentrations of TNF. Reduced Ang-1 and Ang-2 and increased Ang-2/Ang-1 are detected in patients with CM (Conroy et al., 2012; Eisenhut, 2012), which is consistent with the pathophysiological changes of activation and dysfunction of ECs among CM patients. In addition, elevation of specific plasma smooth muscle proteins, including carbonic anhydrase III (CA3), creatine kinase (CK), creatine kinase muscle (CKM), and myoglobin (MB), indicates muscular damage and microvasculature lesions during CM (Bachmann et al., 2014). These proteins may serve as novel biomarkers for predicting CM severity and therapeutic targets for CM.

Previous reports have demonstrated that the expression of circulating microRNAs (miRNAs) is highly sensitive to physiological and pathological stimuli (Paul et al., 2018). As a consequence, their changes in response to *P. falciparum* infection raise the possibility of new diagnostic and potentially prognostic tools for CM. Hsa-miR-3158-3p was found to be effective for the diagnosis of severe/cerebral malaria across all age groups, and hsa-miR-3158-3p represents a promising biomarker candidate for predicting CM prognosis in all age groups (Gupta et al., 2021). In addition, previous studies have shown associations of the relative expression of miR-19a-3p, miR-19b-3p, miR-146a, miR-193b, miR-467a, miR-27a, and miR-146a with CM (Martin-Alonso et al., 2018; Wah et al., 2019; Assis et al., 2020).

Spatial metabolomics is an emerging omics tool that provides precise determination of species, contents, and differential spatial distributions of metabolites in animal/plant tissues (Martin-Alonso et al., 2018; Geier et al., 2020). In the ECM, both kidney and spleen metabolism are differentially perturbed in CM compared with noncerebral malaria, and lipid metabolism and the TCA cycle are altered in the kidney and spleen (Ghosh et al., 2012). Spatial metabolomics is beneficial for the diagnosis, biomarker discovery, and prognosis prediction of CM.

## Treatment

### Antimalarial therapy

Early standard antimalarial treatment is crucial for CM. In 2011, parenteral artesunate was recommended as the first-line treatment for CM by the World Health Organization (WHO). Although artesunate is effective in clearing malaria parasites, administration with potent artemisinin derivatives alone is insufficient to protect against cell death, nerve damage, and cognitive impairment (Brejt and Golightly, 2019), and the long-term and widespread use of artemisinins alone may lead to the emergence of drug-resistant strains. Artemisinin-based combination therapies (ACTs) are therefore introduced to improve clinical outcomes, reduce mortality, prevent long-term neurocognitive deficits and delay the emergence of artemisinin resistance.

### Potential adjuvant therapy

Targeting a single signaling pathway may be insufficient to reduce mortality or improve neurological conditions among CM patients, since CM is a multiprocess disorder. Therefore, adjuvant therapy targeting multiple physiological processes of CM is needed to improve clinical outcomes, prolong survival, and reduce neurological damage in survivors (John et al., 2010). Adjuvant therapy aims to decrease cytoadherence and sequestration, modulate immune responses and improve endothelial functions, with neuroprotection given as a priority, and previous studies have shown the effectiveness of adjuvant therapy in reducing mortality due to CM in ECM models (Wei et al., 2022). However, the results from clinical trials are disappointing.

#### Targeting parasite adhesion to vascular endothelium

Clinical episodes of CM are associated with the expression of *var* genes encoding the specific PfEMP1 protein, while *Var* genes are independently observed to bind to the brain endothelium *in vitro* (Avril et al., 2012; Claessens et al., 2012). Once the crucial *var* ligand and its endothelial receptors are identified, high-throughput screening may be used to identify small molecules that block the binding or activation of microvascular endothelium by iRBCs. Levamisole was found to interrupt CD36-dependent binding by inhibiting CD36 dephosphorylation, which is required for high-affinity binding (Miller et al., 2013). It is therefore suggested that blockade of malaria parasite adhesion to the vascular endothelium may be a promising strategy for CM treatment.

#### Regulating immune responses

Preventive measures prior to malaria may alter the immune system status and delay CM development; therefore, adjuvant therapy targeting immune regulation is difficult. Previous animal studies have identified modulators of host targets as potential adjuvant therapies, opening up new avenues for developing highly selective adjuvant therapies for CM. Targeting mammalian targets of rapamycin (mTOR) with rapamycin has been proven to be effective in suppressing immune responses (Mejia et al., 2015), thus supporting the potential of rapamycin as an adjuvant treatment for CM. 6-Diazo-5-oxo-L-norleucine (DON), a glutamine analog, was found to block the glutaminase-mediated conversion of glutamine to glutamate, thereby inhibiting T-cell activation (Crunkhorn, 2015), and administration of DON resulted in survival from CM and brain recovery in ECM (Gordon et al., 2015). These data demonstrate that regulation of immune balance may be effective for CM treatment.

#### Improving endothelial functions and maintaining endothelial barrier integrity

Several therapeutics have been found to target endothelial dysfunction, including a platelet-activating factor receptor antagonist (Lacerda-Queiroz et al., 2012), statins such as atorvastatin (Souraud et al., 2012) and lovastatin (Reis et al., 2012), activated protein C (Mohan Rao et al., 2014), and erythropoietin (Kaiser et al., 2006). In addition, Ang protein was reported to regulate endothelial barrier integrity and is associated with CM-induced retinopathy and death (Conroy et al., 2012). In response to TNF stimulation, Ang-2 causes destruction of endothelial barrier integrity and triggers endothelial adhesion molecule expression. Secretion of Ang-2 in endothelial Weibel-Palade bodies may lead to vascular leakage, inflammation, and encephaledema associated with CM. Endothelium-targeted therapy that inhibits Weibel-Palade extracellular secretion may block the pathogenic autocrine activity of Ang-2 (Yeo et al., 2008).

#### Neuroprotection

CM is a severe neurological syndrome that may cause epilepsy, coma and death, and survivors may present with neurological and cognitive deficits. Protection of nerve cells is therefore highly essential. Among the potential neuroprotective agents, erythropoietin (EPO) is one of the most promising. In addition to stimulating erythropoiesis, EPO has neuroprotective functions and increases the stability of endothelial barriers (Ghezzi and Brines, 2004; Maiese et al., 2005). Artesunate plus recombinant human erythropoietin (rhEPO) has been found to reduce endothelial activation and improve BBB integrity in murine ECM models, resulting in faster recovery, increased survival rates, and high neuroprotective effects (Du et al., 2017). Administration of peroxisome proliferator-activated receptor-gamma (PPARγ) has been proven to improve long-term cognitive ability and prolong survival (Serghides et al., 2014). In addition, PPARγ has shown neuroprotective effects *via* various pathways and promotes neuronal repair, making it an attractive adjuvant therapy. Dysregulation of the limk-1/cofilin-1 pathway might lead to alterations in neuronal morphology and is considered the cause of cognitive defects in patients surviving CM (Simhadri et al., 2017); therefore, the LIMK-1/cofilin-1 pathway is considered a potential therapeutic target for CM. In addition, granzyme-B produced by CD8^+^ T cells directly kills neurons through cytotoxic function and activation of caspase-3 and calpain1 (Kaminski et al., 2019). Therefore, targeting granzyme-B may be an option to prevent neuronal cell death.

Unfortunately, the clinical efficacy and safety of these adjuvant treatments have not been tested until now. Inclusion of specific PfEMP-1 receptors on the surface of iRBCs may allow its connection with T cells to yield the ability to kill iRBCs, thus inhibiting the downstream pathological reactions initiated by iRBC adhesion. Chimeric antigen receptor T (CAR-T) immune cell therapy is a breakthrough for cancer therapy (Herzig et al., 2019; Bertoletti and Tan, 2020). Since iRBC adhesion is the initial step during the development of CM, the efficacy and safety of CAR-T immune cell therapy for CM deserve further investigation.

## Conclusions and perspectives

CM is a multifactorial and multiprocess disorder. Administration of antimalarials alone is effective in clearing malaria parasites; however, such a treatment fails to protect against nerve cell death, neurological damage and cognitive impairment. This urges the development of novel treatment for improved outcomes of CM. In addition, the rapid developments of -omics offer an opportunity for understanding the etiology of CM and provide insights into the clinical diagnosis and therapy of this potentially fatal disorder.

## Author contributions

JL, HD, and WWe conceived and designed the study. XS, WW, WC, HZ, WWa, HD, and JL wrote the paper. All the authors read and approved the final manuscript.

## Funding

This study was supported by the National Natural Science Foundation of China (Grant Number 81802046), the Principle Investigator Program of Hubei University of Medicine (Grant Number HBMUPI202101) and the Advantages Discipline Group (Public health) Project in Higher Education of Hubei Province (2022PHXKQ1).

## Conflict of Interest

The authors declare that the research was conducted in the absence of any commercial or financial relationships that could be construed as a potential conflict of interest.

## Publisher’s note

All claims expressed in this article are solely those of the authors and do not necessarily represent those of their affiliated organizations, or those of the publisher, the editors and the reviewers. Any product that may be evaluated in this article, or claim that may be made by its manufacturer, is not guaranteed or endorsed by the publisher.
